# Data evidencing slow anaerobic digestion in emergency treatment and disposal of infectious animal carcasses

**DOI:** 10.1016/j.dib.2018.12.001

**Published:** 2018-12-06

**Authors:** Jacek A. Koziel, Heekwon Ahn, Thomas D. Glanville, Timothy S. Frana, J. (Hans) van Leeuwen, Lam T. Nguyen

**Affiliations:** a Department of Agricultural and Biosystems Engineering, Iowa State University, Ames, IA 50011, USA; b Department of Civil, Construction and Environmental Engineering, Iowa State University, Ames, IA 50011, USA; c Department of Food Science and Human Nutrition, Iowa State University, Iowa State University, Ames, IA 50011, USA; d Department of Animal Biosystems Science, Chungnam National University, Daejeon, Republic of Korea; e Department of Veterinary Diagnostic and Production Animal Medicine, Iowa State University, Ames, IA, USA

## Abstract

Burial of infectious and potentially infectious livestock and poultry animals is the most common response to an emergency situation. The data set summarizes 22-week-long experiment that simulates the environment found within conventional burial trenches for emergency disposal of animal carcasses, worldwide, sometimes with a topical application of quicklime as it is required in the Republic of Korea. This data set shows the rarely presented evidence of the extremely slow decay of animal carcasses. Besides visual evidence of no visible breakdown of carcass material, i.e., carcass (or carcass quarters and coarse cuts) still resembled the initial material at the end of the study, we present data characterizing the process. Specifically, temporal variations of digestate quality (pH, ammonia, volatile fatty acids), biogas production, and the persistence of odorous volatile organic compounds are summarized. The data provide important evidence of undesirable, slow progression of the digestion process. The evidence of failure to achieve practical endpoints with the anaerobic digestion provides the impetus for seeking alternative, improved methods of disposal that will be feasible in emergency context, such as aerated burial concept (Koziel et al., 2018 [1]).

**Specifications table**

**Table:** 

Subject area	*Engineering, Agricultural and Biological Sciences, Livestock Production Systems*
More specific subject area	*Waste management, animal carcass disposal*
Type of data	*Images, figures*
How data was acquired	*Gas chromatography - mass spectrometry (GC–MS), temperature and pH probes*
Data format	*Analyzed data*
Experimental factors	*Decays of the whole, chopped poultry carcasses with/without the addition of quicklime were evaluated by measuring key operating variables. These variables were needed as an ultimate proof of process’ extreme slow rate and the lack of apparent decay progress.*
Experimental features	*22-week long anaerobic digestion of whole and chopped poultry carcasses simulating disposal of diseases mortalities in an in-trench burial.*
Data source location	*Department of Agricultural and Biosystems Engineering at Iowa State University, Ames, Iowa, USA.*
Data accessibility	Data is within this article.
Related research article	Koziel, J.A., H.K. Ahn, T.D. Glanville, T.S. Frana, H. van Leeuwen, L.T. Nguyen. 2018. Lab-scale evaluation of aerated burial concept for treatment and emergency disposal of infectious animal carcasses. *Waste Management*, 76, 715–726. doi:10.1016/j.wasman.2018.03.009[1].

**Value of the data**

• The digestate quality data summarizing temporal variations pH, ammonia, volatile fatty acids were compared with the optimal range recommended for anaerobic digestion. Comparing and reflecting on these operating parameters can help to explain the apparent lack of carcass decay.
• The data could be used to design an improved, more successful emergency carcass disposal that must relay of an in-trench burial with anaerobic treatment. The in-trench burial is the most common worldwide approach for emergency carcass disposal.
• The data documents ranges of design parameters (pH, ammonia, and volatile fatty acids) that describe the environment of a common in-trench burial. In-trench burial environment that can be very different from typical anaerobic digestion operations used for waste management on a municipal or industrial scales.
• The data can be also used for estimating costs of implementing various physical and chemical pre-treatments and interventions that would be needed to adjust the in-trench carcass burial environments into the range of operating parameters associated with the industrial or municipal scale anaerobic digestion process. Such estimations are needed to determine if proposed solutions are practical during outbreaks of infectious livestock or poultry diseases.
• The evidence of failure to achieve apparent practical carcass decay with the anaerobic digestion provides the impetus for seeking alternative, improved methods of disposal that will be feasible in emergency situation context, such as aerated burial concept for accelerated digestion [1], [2], [3].

## 1 Data

This section summarizes the key operating parameters in a 22-week long anaerobic treatment of poultry carcasses:

• measured pH levels,
• volatile fatty acids concentrations,
• ammonia concentrations,
• bulk biogas production rates, and the
• evidence of the persistent presence of offensive odorous volatile organic compounds (VOCs) in reactor headspace and the lack of odor mitigation.

**Fig. 1:**
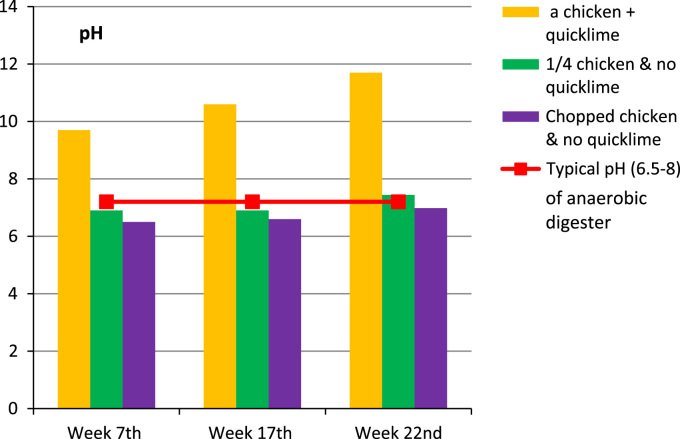
Anaerobic carcass disposal: measured pH in digestate.

**Fig. 2:**
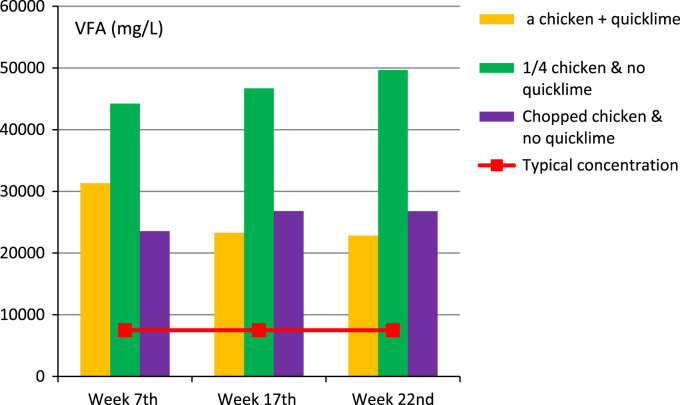
Anaerobic carcass disposal: volatile fatty acid concentrations in digestate.

**Fig. 3:**
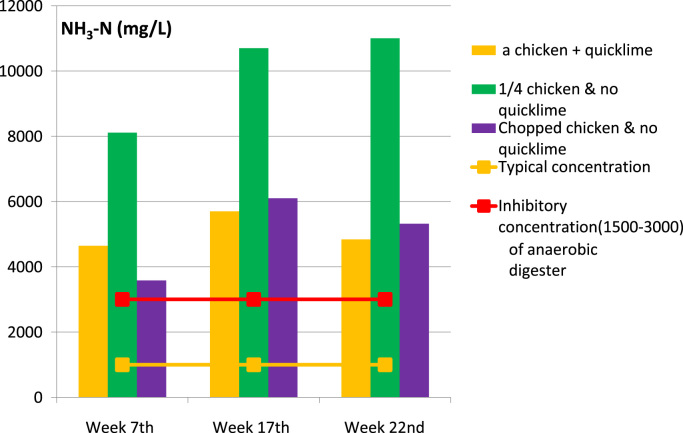
Anaerobic carcass disposal: measured ammonia in digestate.

The assessment of digestate quality summarizes pH (Fig. 1), volatile fatty acids (Fig. 2), and ammonium (Fig. 3) allows for comparison with the optimal range recommended for industrial processes involving anaerobic digestion. Data summaries for these resulting parameters describing in-trench burial environments can help to explain apparent slow carcass decay.

Free ammonia (generally considered bactericidal) dominates at pH>pKa, which was the case in the reactor with added quicklime (Fig. 3). Measured ammonia concentrations were well above (bacterial) inhibitory concentrations (Fig. 3).

**Fig. 4:**
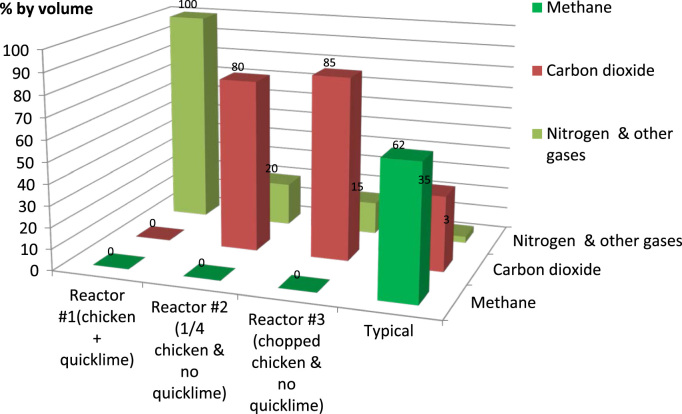
Anaerobic carcass disposal: bulk biogas composition.

There was no measurable bulk biogas (CO_2_, CH_4_) production (Fig. 4).

**Fig. 5:**
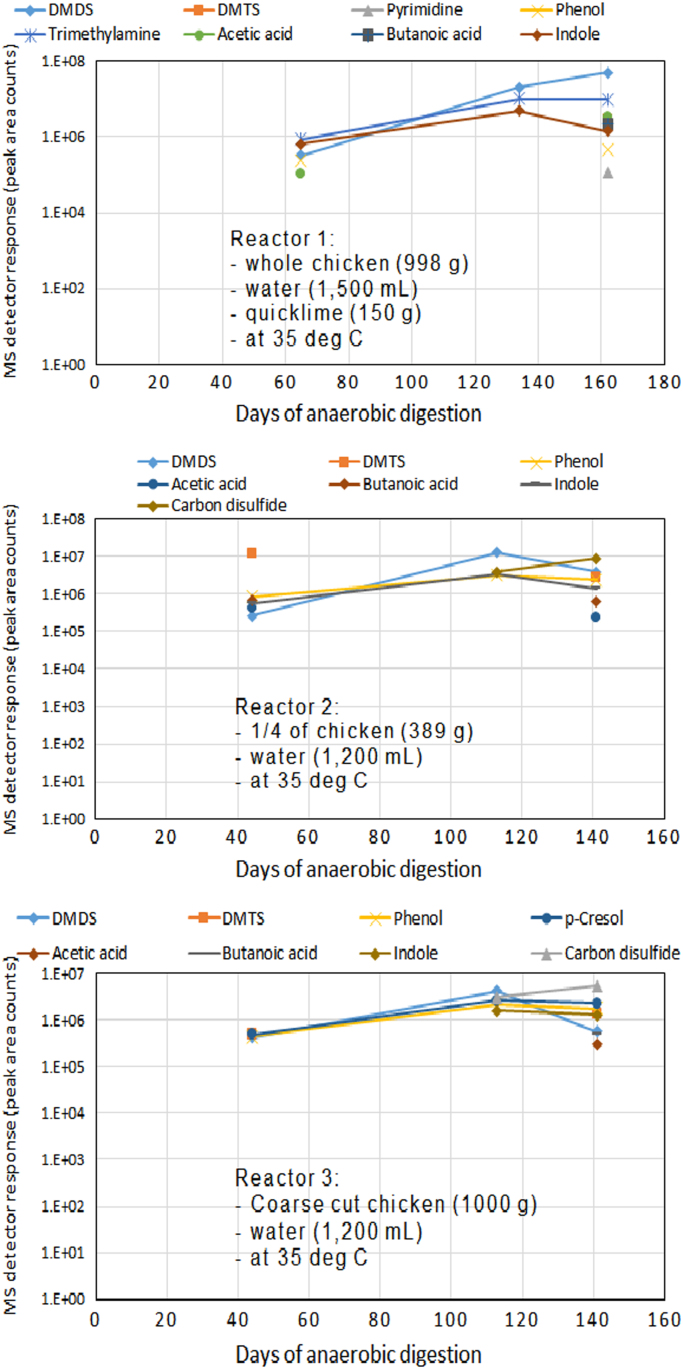
Anaerobic carcass disposal: evidence of the persistent presence of offensive odorous VOCs in reactor headspace and the lack of odor mitigation. DMDS=dimethyl disulfide; DMTS=dimethyl trisulfide.

VOCs identified in the reactors headspace were offensive odorants (Fig. 5) many of which increased in concentration concomitant with treatment.

## 2 Experimental design, materials, and methods

**Fig. 6:**
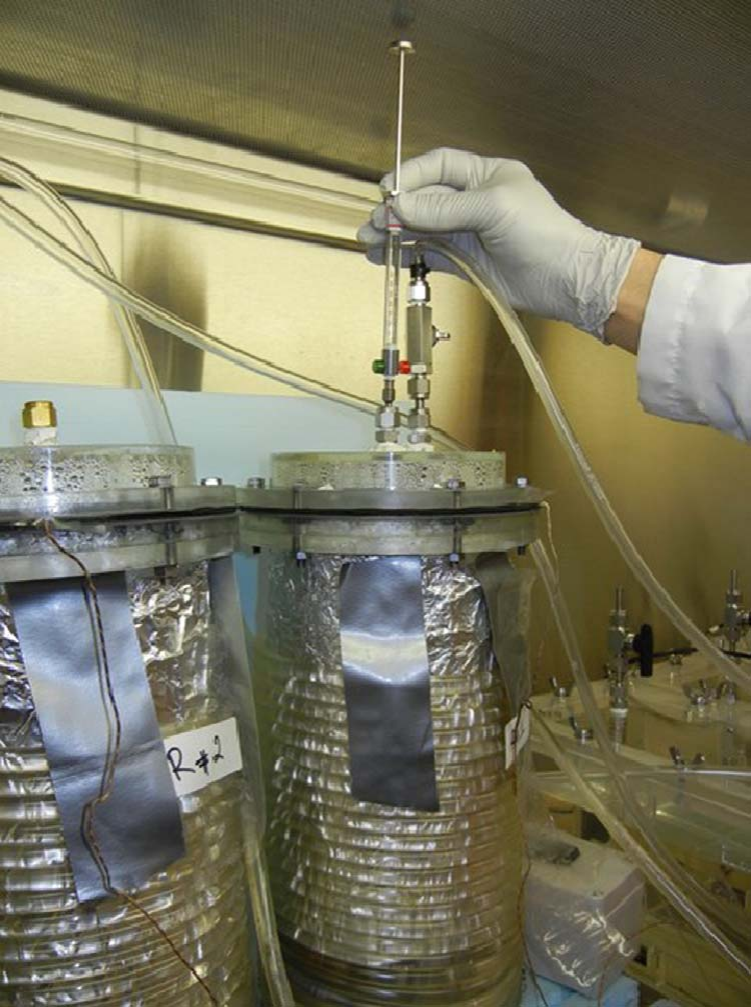
22 week-long trial of anaerobic poultry carcass disposal in 6 L reactors: collection of biogas samples.

The experiments were conducted over 22-week-long trial using reactors simulating anaerobic digestion that is typical in conventional burial trenches. Some countries (e.g., the Republic of Korea) recommend or require a surficial application of quicklime (CaO) as a means to mitigate odor and pathogens. Materials and methods are described in greater detail elsewhere [1]. Briefly, the reactors were constructed from 0.154 m diameter × 0.305 m tall Plexiglas cylinder, with sealed top and bottom (Fig. 6) fitted with ports for the collection of liquid and gas samples. Three types of treatments were used:

• (Reactor 1) whole poultry carcass (998 g, whole chicken) with quicklime added (150 g),
• (Reactor 2) one quarter (1/4) portioned of whole poultry carcass (389 g), no quicklime,
• (Reactor 3) coarse cuts of whole poultry carcass (1000 g of total mass, chopped chicken), no quicklime.

Approximately 1.2 to 1.5 L of water was added to each reactor to simulate a burial trench environment containing whole and partially decayed carcasses and the digestate. The reactor temperature was maintained at 35 °C (Fig. 6). The translucent tops of reactor allowed for periodic visual inspection of the digestion process and the apparent lack of visible breakdown of carcass material, even at the end of the experiment. Reactors were housed inside a biosafety cabinet to lower the risk of accidental spread of potentially infectious pathogens and an accidental release of odorous gases.

Measurements of pH, volatile fatty acids and ammonia in digestate were measured on week 7, 17, and 22. Measurements of volatile organic compounds (VOCs) in reactor headspace were conducted on week 8, 19, and 23 (Reactor 1), and on week 6, 16, and 20 (Reactors 2 and 3). Headspace gas was collected using solid-phase microextraction (SPME) and analyzed using gas chromatography – mass spectrometry (CG-MS). Data were analyzed qualitatively by comparing MS detector response to VOCs abundance measured by peak area counts. VOC sampling conditions for SPME were identical for all reactors. Biogas was collected from reactor syringe using 1 mL Pressure-Lok gas syringe and analyzed on a CG-FID-ECD.
