# Supplementary material for: Data evidencing slow anaerobic digestion in emergency treatment and disposal of infectious animal carcasses
Source: Data Brief. 2018 Dec 6;22:227–33. doi: 10.1016/j.dib.2018.12.001 (PMC6302243; doi:10.1016/j.dib.2018.12.001)
Supplement: Supplementary file 1 — Supplementary material. [file mmc1.docx]

AUTHOR DECLARATION

We wish to confirm that there are no known conflicts of interest associated with this publication.

On behalf of all co-authors:

Dr. Jacek Koziel, Prof.

Dept. of Agricultural & Biosystems Engineering

4350 Elings Hall, Iowa State University, Ames, IA 50011, USA

515-294-4206. [koziel@iastate.edu.](mailto:koziel@iastate.edu)
